# Avian Influenza A (H7N9) and related Internet search query data in China

**DOI:** 10.1038/s41598-019-46898-y

**Published:** 2019-07-18

**Authors:** Ying Chen, Yuzhou Zhang, Zhiwei Xu, Xuanzhuo Wang, Jiahai Lu, Wenbiao Hu

**Affiliations:** 1 School of Public Health, Sun Yat-sen University, Guangzhou, China; 2 School of Public Health and Social Work, Institute of Health and Biomedical Innovation, Queensland University of Technology, Brisbane, Australia

**Keywords:** Preventive medicine, Epidemiology, Risk factors

## Abstract

The use of Internet-based systems for infectious disease surveillance has been increasingly explored in recent years. However, few studies have used Internet search query or social media data to monitor spatial and temporal trends of avian influenza in China. This study investigated the potential of using search query and social media data in detecting and monitoring avian influenza A (H7N9) cases in humans in China. We collected weekly data on laboratory-confirmed H7N9 cases in humans, as well as H7N9-related Baidu Search Index (BSI) and Weibo Posting Index (WPI) data in China from 2013 to 2017, to explore the spatial and temporal trends of H7N9 cases and H7N9-related Internet search queries. Our findings showed a positive relationship of H7N9 cases with BSI and WPI search queries spatially and temporally. The outbreak threshold time and peak time of H7N9-related BSI and WPI searches preceded H7N9 cases in most years. Seasonal autoregressive integrated moving average (SARIMA) models with BSI (*β* = 0.008, *p* < 0.001) and WPI (*β* = 0.002, *p* = 0.036) were used to predict the number of H7N9 cases. Regression tree model analysis showed that the average H7N9 cases increased by over 2.4-fold (26.8/11) when BSI for H7N9 was >  = 11524. Both BSI and WPI data could be used as indicators to develop an early warning system for H7N9 outbreaks in the future.

## Introduction

Avian influenza A (H7N9) has posed a threat to public health in China in recent years^1^. Human cases of H7N9 have had a winter-spring peak with high mortality rates (range: 34–47%) in the past five annual epidemics^2^. After the first H7H9 epidemic began in March 2013, the number of cases gradually decreased during the next three epidemics cycles. However, the epidemic in 2016–2017 was worse with 766 laboratory-confirmed cases observed, which accounted for 49.1% (766/1560) of total H7N9 cases reported from 2013–2017. The median incubation period of H7N9 cases (from exposure to disease onset) was six days^3^ and the time from onset to disease confirmation was seven days^4^. After confirmation, the national health system receives the report from the hospital and then carries out measures to control epidemics. Therefore, traditional surveillance systems have a time lag of about two weeks. To prepare for the next epidemic and to provide a timely and effective response, a new approach for achieving near real-time detection of H7N9 cases and even prediction of emerging and spreading infectious outbreaks should be developed. Internet-based surveillance has the potential to achieve these goals.

The last decade has seen the rapid emergence of big data and data science research, which relies on the increasing availability of electronic records generated by using the Internet, mobile phones, and satellites, etc^5^. These non-traditional digital data sources include social media, web search engines, and remote sensing. Internet-based disease surveillance has been widely suggested as a potential means to improve infectious disease surveillance^6^. Several infectious disease surveillance systems have been developed using internet search metrics to estimate incidence, including for influenza^7,8^ (Google Flu Trends) and dengue^9^ (Google Dengue Trends)^10^. Internet search metrics have also been applied in the monitoring and forecasting of emerging and re-emerging infections, including pandemic Ebola^11^ and Zika^12^, etc.

The number of China’s internet users reached 772 million at the end of 2017 and it is still increasing^13^. Baidu is the most widely used search engine in China (approximately 86.7% of internet users in China use Baidu^14^), and Weibo is the most popular social media site in China^15^. Due to insufficient knowledge of disease symptoms and transmission mechanisms^16^, people may search H7N9-related information online prior to H7N9 season. The variety of online activity could provide additional data sources to public health authorities and governments for detecting and monitoring H7N9 in a timely manner.

Few studies have been done to quantify the relationship between Internet search query data and H7N9 infection. This study aims to (1) explore the temporal and spatial trends of laboratory-confirmed H7N9 cases in humans; (2) screen the use of H7N9-related keywords in search engine and social media data; (3) assess the relationship of H7N9 case numbers with H7N9-related search and internet posting indices; and (4) provide useful information for developing an avian influenza early warning system using big data.

## Results

### Temporal distributions of influenza A (H7N9) Human Cases and Baidu Search Index (BSI) and Weibo Post Index (WPI) search data for H7N9

**Figure 1:**
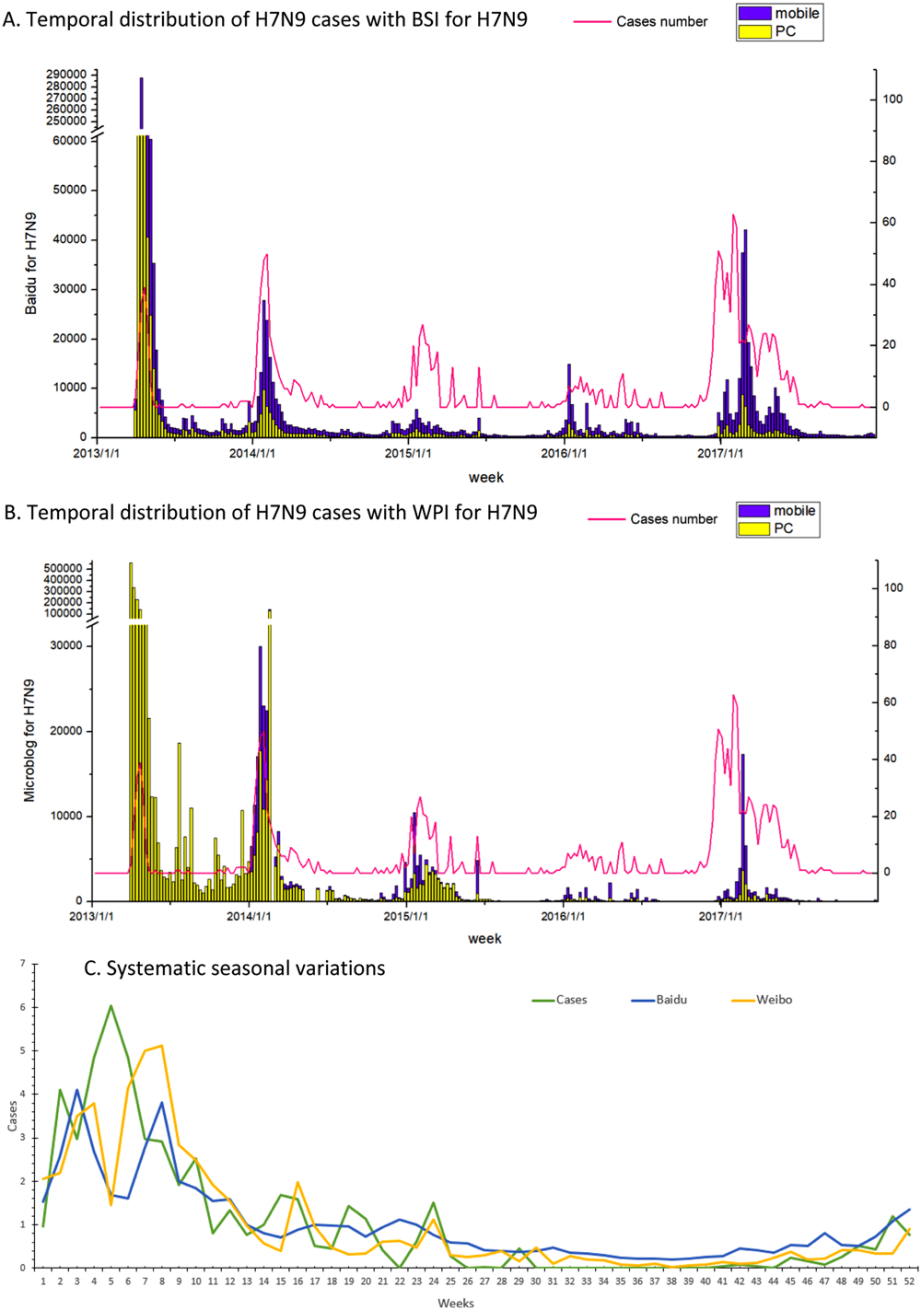
The temporal distribution of Avian influenza A (H7N9) confirmed human cases and Baidu Searching Index (BSI) and Weibo Post Index (WPI) search data for search term “H7N9” in China from 2013–2017. Note: PC indicates personal computer in panel A and B; week 1 indicates the first week of the calendar year (the week containing January 1) in panel C.

From March 2013 to December 2017, a total of 1,560 laboratory-confirmed H7N9 cases were reported in China, with H7N9 cases being reported in 11 provinces in the wave 1 and 29 provinces in wave 5. The mean values of weekly BSI for search terms “H7N9”, “avian influenza” and “live poultry” were 5,758.5, 7,429.2, and 5,497.8, respectively. The mean values of weekly WPI for the three keywords “H7N9”, “avian influenza” and “live poultry” were 9601.9, 561.1, and 6017.4, respectively. Temporal trends of BSI and WPI for search term “H7N9” and H7N9 cases number are illustrated in Fig. 1, panel A and panel B. H7N9 case number, BSI, and WPI all peaked between December to February and troughed from April to October (Fig. 1, panel C).

### Spatial distributions of Influenza A (H7N9) human cases and Baidu Searching index (BSI) search data for H7N9

**Figure 2:**
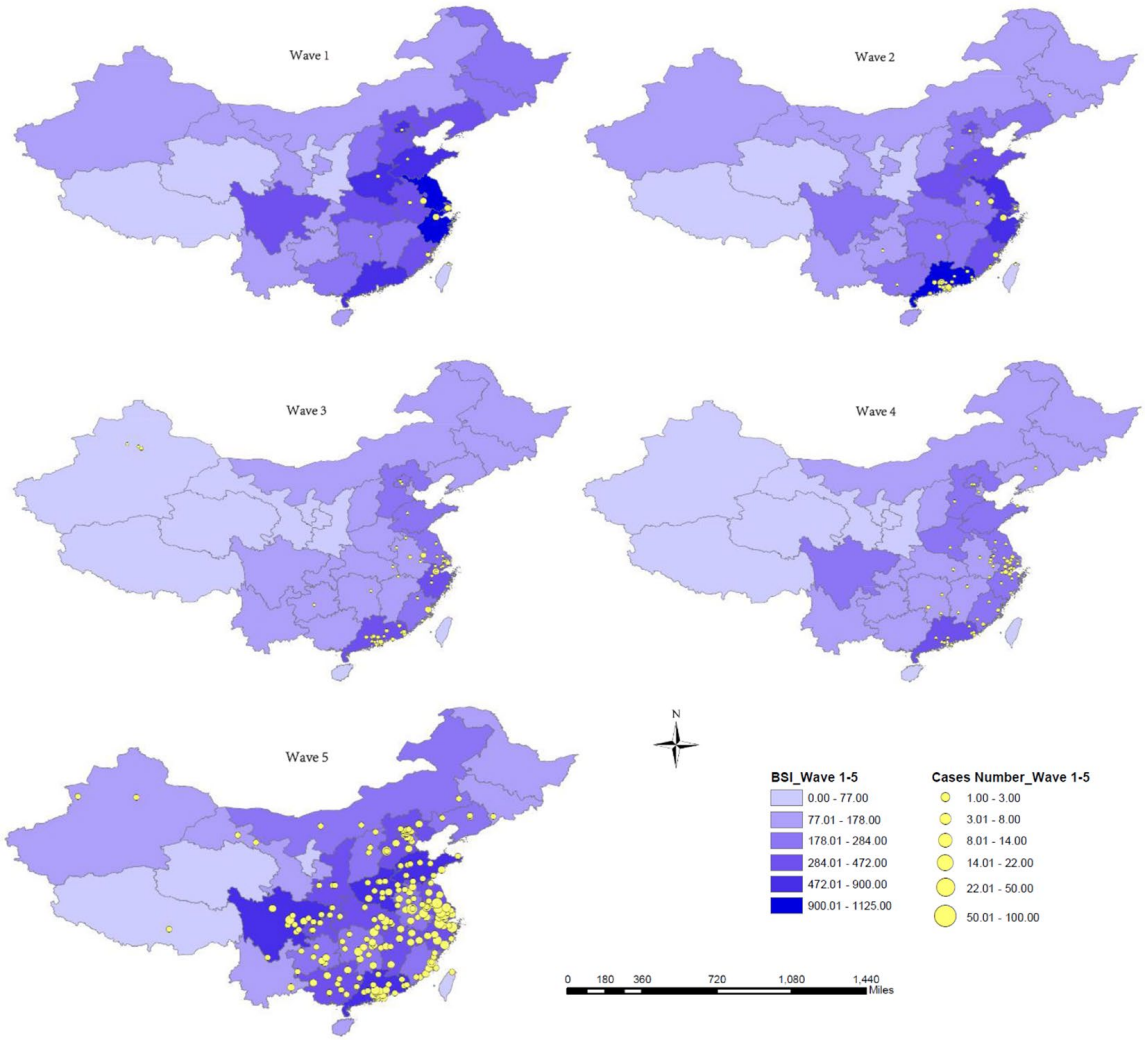
Spatial distribution of Avian influenza A (H7N9) confirmed cases and Baidu Searching Index (BSI) search data for H7N9 in China from 2013–2017. Note: Wave 1 was from Jan 1st to Sept 30th, 2013, wave 2 was from Oct 1st, 2013 to Sept 30th of 2014, wave 3 was from Oct 1st, 2014 to Sept 30th of 2015, wave 4 was from Oct 1st, 2015 to Sept 30th of 2016, and wave 5 was from Oct 1st, 2016 to Dec 31st, 2017.

Figure 2 shows that H7N9 cases and BSI for search term “H7N9” during wave 1 were mainly concentrated in the Yangtze River Delta area. For waves 2 to 4, H7N9 cases and BSI for “H7N9” spread to include eastern China and southern China (Pearl River Delta area). For wave 5, H7N9 cases and BSI for “H7N9” spread across almost the whole country except the far west and north east. The Poisson log-linear regression model shows the H7N9 case and BSI spatial dispersion (change in longitude and latitude) in each of the epidemic waves (Table S1). The positive coefficients for longitude (eastward movement) and the negative coefficients for latitude (southward movement) indicate general consistency but some differences in the extent and direction of cases and BSI special dispersion in each of the five epidemic waves.

### BSI and WPI from two type platforms

The temporal and spatial trends in searching and posting of the term “H7N9” using personal computers (PC) and mobile devices are shown in Fig. S1. Figure S1A shows that the temporal trends in the proportion of “H7N9” term searching and posting using mobile devices increased year by year, especially for Weibo. Figure S1B displays the provinces sorted by mobile usage ratio of BSI for H7N9 from high to low over the 5 year period. A linear regression model was then built by the sequence of mobile usage ratio and GDP ranking of provinces (Fig. S1C)^17^. The negative coefficient indicates that the lower GDP regions had a higher proportion of mobile device usage.

### Comparison of outbreak duration

The identified outbreak start-time and the peak time of BSI and WPI H7N9-related searches always preceded the H7N9 case outbreak start time and peak time in waves 1–4. Moreover, the outbreak duration of BSI was longer than the case outbreak duration by 2–8 weeks, and the outbreak duration of WPI was longer than the case outbreak duration by 3–10 weeks (Table S2).

### Time-series cross-correlation analysis

**Figure 3:**
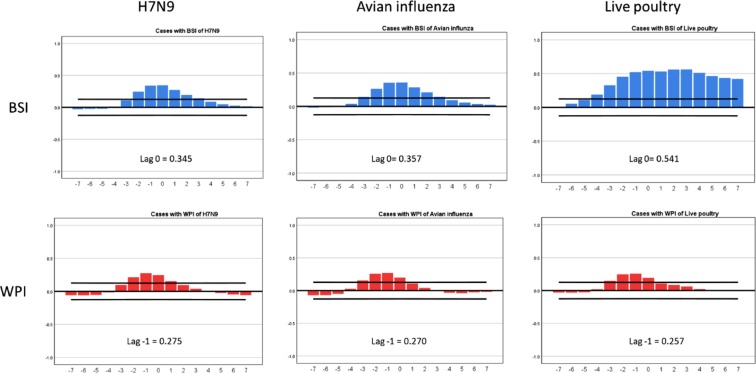
Cross-correlation between H7N9 cases with BSI and WPI for searching and posting the H7N9, Avian influenza and Live poultry. Note: Blue bars indicate the value of BSI data from 2013–2017. The value of WPI data is displayed by red bars. Confidence intervals (95%) are indicated by the black line (X axis: lag value; Y axis: CCF value, defined as the set of sample correlations; highest value of lag and CCF are marked in each panel).

The time-series cross-correlation analysis (Fig. 3) demonstrates that weekly H7N9 case occurrence was positively correlated with weekly BSI with a lag of −2 to +3 weeks for the search term “H7N9”, a lag of −3 to +3 weeks for the search term “Avian influenza”, and a lag of −4 to +7 weeks for the search term “Live poultry”. Weekly H7N9 case occurrence was positively correlated with weekly WPI with a lag of −2 to +1 weeks for the search term “H7N9”, with a lag of −3 to 0 weeks for “Avian influenza”, and a lag of −3 to 0 weeks for “Live poultry”. Among the three search keywords, “Live poultry” of BSI had the highest lead time (−4) compared with H7N9 cases. The strongest correlation between cases and indices, for BSI, was at a lag of 0 week, and for WPI was at a lag of −1 week.

### The diagnostic tests of each search terms for predicting the number of H7N9 cases

**Figure 4:**
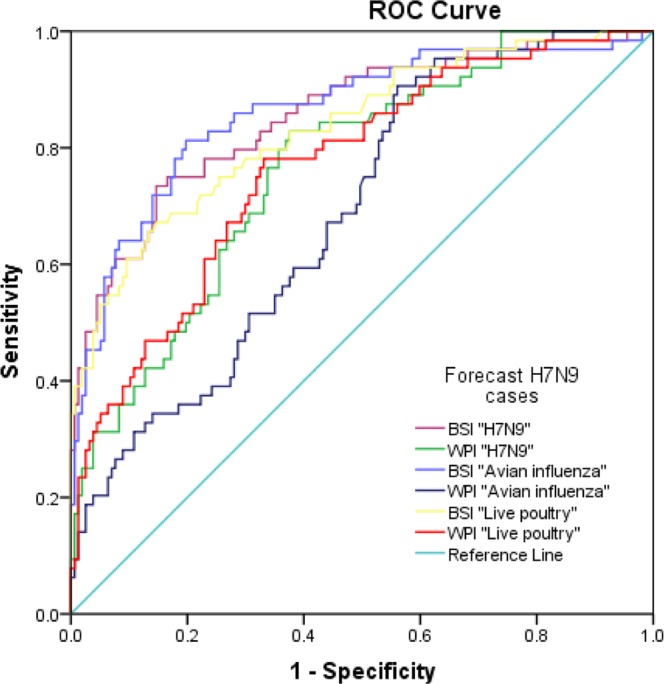
The ROC (receiver operating characteristic) curves for prediction of H7N9 cases using BSI (Baidu search index) and WPI (Weibo post index) for searching and posting the H7N9, Avian influenza and Live poultry. Note: Area under the curve: BSI “Avian influenza”, 0.861; BSI “H7N9”, 0.855; BSI “live poultry”, 0.834; WPI “Avian influenza”, 0.767; WPI “H7N9”, 0.765; WPI “live poultry”, 0.687.

A receiver operating characteristic (ROC) analysis was performed to assess the sensitivity and specificity of BSI and WPI with three search terms, generating a good prediction of H7N9 epidemics at the threshold of annual average reported number of H7N9 cases. The area under the receiver operating characteristic curves (AUC) of six platform-search term indices ranged from 0.687 to 0.861 (Fig. 4). The AUCs of BSI indices were higher than the AUCs of WPI.

### Seasonal Autoregressive integrated moving average (SARIMA) model

SARIMA models with BSI and WPI as independent variables were built to predict the occurrence of H7N9 cases. Separate SARIMA models with BSI and WPI for search term “H7N9” were developed to forecast H7N9 case numbers. The SARIMA model (4, 0, 0) (4, 0, 0) and (1, 0, 0) (1, 0, 0) using BSI and WPI were found to fit the data well. Furthermore, the (1, 0, 0) (1, 0, 0) was the best parameter of (p, d, q) to fit the forecast model for the BSI and WPI with H7N9 cases number (*P* < 0.05). The increased R^2^ (0.736 versus 0.748 and 0.740, respectively) and decreased BIC value (3.585 vs 3.550 and 3.581, respectively) indicated the BSI and WPI can forecast the H7N9 infection and improve the effect of predictive model (Table S3, Figs S2 and S3).

### Regression tree model

**Figure 5:**
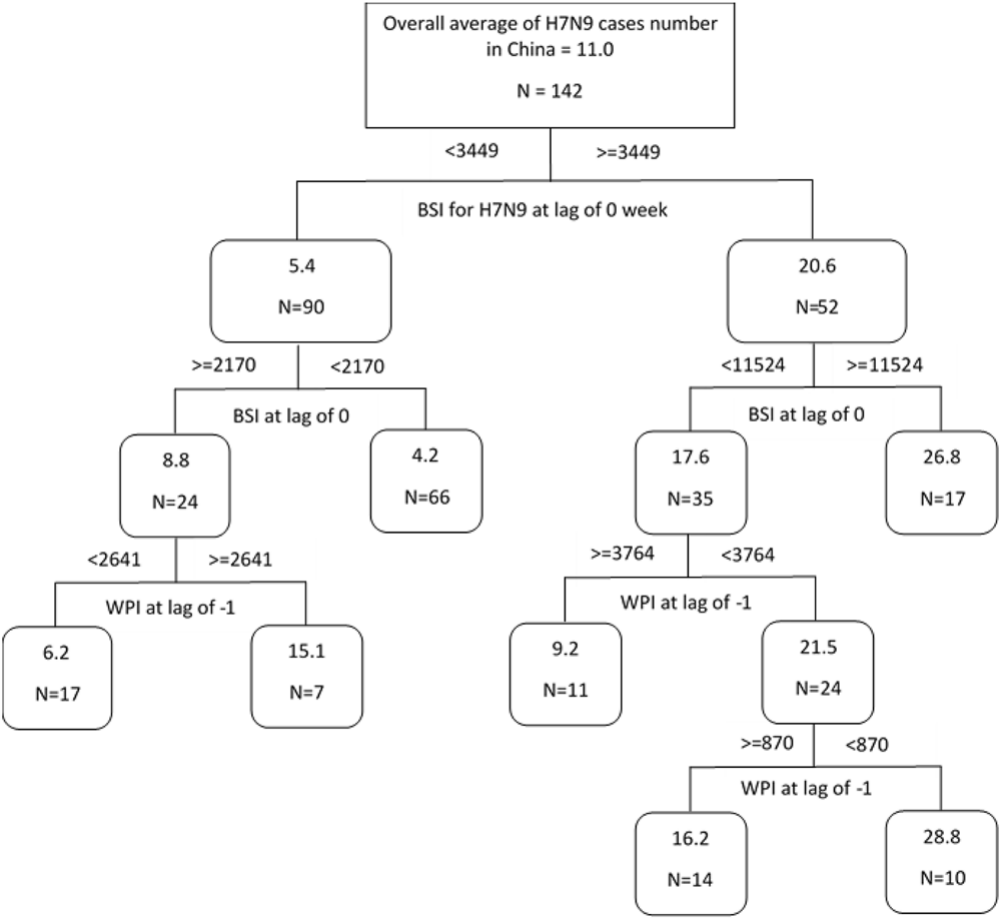
The regression tree modelling the hierarchical relationship between weekly H7N9 cases number with Baidu Searching Index (BSI) and Weibo Posting Index (WPI) in China from 2013 to 2017. Note: The numbers in boxes shown the average of weekly H7N9 cases number and N that is the total week count of occurrence of H7N9 cases, the numbers out of boxes shown threshold values generated by regression tree.

We used the BSI with a lag of 0 week and WPI with a lag of −1 week for “H7N9” to build a regression tree. Figure 5 demonstrates that the 0-week lagged BSI was the first level factor in the model, and the lagged WPI was the second level factor. When BSI for H7N9 at 0-week lag was >=11524, the average H7N9 cases increased by over 2.4-fold (26.8/11). The average H7N9 cases increased by over 2.6-fold (28.8/11) when WSI for H7N9 at -1-week lag was < 870.

## Discussion

Online search queries are a uniquely valuable source of historical and real-time information. The potential of using Internet-based data to improve traditional infectious disease surveillance has been increasingly explored in recent decades^9^, especially for vaccine-preventable and vector-borne diseases^10^. However, previous research has been mainly conducted in industrialized and highly information intensive countries^18^; the situation in developing countries or areas with poorer socioeconomic levels is unknown.

Our study has shown positive spatial and temporal relationships of H7N9 cases in humans with H7N9-related search engine data from BSI and WPI. Unlike most current research, which reflects the temporal correlation of disease with Internet-based data^19^, our study also focuses on the spatial relationship between Internet-based data and disease. Our study found that people pay attention to and search disease-related vocabulary as the scope of disease spread changes, which may be less affected by network popularity and economic level. Using geographic resources such as HealthMap can aid in the timely assessment of risk factors, improve the effectiveness of intervention measures and the accuracy, sensitivity, and visualization of the disease surveillance system^20^.

The results of cross-correlation can indicate how much in advance can Internet-based data give a warning of disease outbreaks. Our findings show that the consistently negative lag values indicate that the WPI index could be an early warning indicator for H7N9 cases. The BSI of search term “live poultry” can be present and be increasing four weeks before the epidemic occurs, providing adequate time for government and health authorities to implement preventive measures. We’ve also observed that the duration of the outbreak and the peak time of BSI and WPI for H7N9-related search terms preceded the occurrence of H7N9 cases in most epidemic waves, suggesting that BSI and WPI are potential indicators for the magnitude and duration of H7N9 epidemics. The reason why the beginning of the outbreak as indicated in BSI and WPI data during the fifth wave (2016–2017) did not occur before that H7N9 cases might be due to the over-reporting by the media or the fear-based search for the cases outbreak^21^. The positive and negative lags in the cross-correlation analyses implied that public opinion can also be affected by disease outbreaks. Therefore, a combination of search engine index and social media index has the potential to be used for both disease surveillance and public opinion monitoring^22^.

Moreover, the results of SARIMA model also suggest that indices of search engine and social media searches can provide additional data for the H7N9 surveillance, and the results showed the good predictive capacity of that model as well. The AUC of the ROC curve indicated that the sensitivity and specificity of BSI were higher than that of WPI. This may possibly be because compared with posting an opinion or discussion on the internet, more people may search for related information about the disease, which may enhance the sensitivity of the BSI. Other studies have used search engine data rather than social media data. However, our study also showed that data from social media, like Weibo, can improve the ability to predict H7N9 human infection. The regression tree model identified that the 0-week lagged BSI for H7N9 was a key predictor of the occurrence of H7N9 in China. A possible reason is that search engines are more popular and indispensable with the relatively unbiased user groups^23^. When the BSI volume is less than around 2000 searches per week, it can be defined as a non-epidemic, baseline period for H7N9 infection, more than 3000 is a warning period, and more than 10000 can be regarded as an outbreak period. This threshold provides an index reference to assess the potential risk of an outbreak based on to peoples’ behaviour on the Internet and to predict the number of possible H7N9 cases.

“H7N9”, “Avian influenza” and “Live poultry” were identified as the most relevant and sensitive search terms for H7N9 infection on the Baidu and Weibo platforms. The transmission of avian flu can be affected by many factors, and live poultry infection is one of the crucial links for human infection. The live poultry markets have been closed in many high-incidence areas for H7N9^24^. Most people who want to buy live poultry mainly search for “live poultry” instead of “H7N9”, which may make it an important indicator for big data analyses for avian influenza. Therefore, different from other influenza subtypes, it is important to consider using “live poultry” as a search term to gather online information on H7N9.

From our study, it can be seen that the usage rate of the mobile phones for both Baidu and Weibo increased to around 70% in recent years, indicating that mobile phones will become the main channel to obtain information online, especially for those socioeconomically disadvantaged areas^25^. In the future, the dissemination of health information and health education may reach a wider range of people through mobile phones than through other means. The higher usage of mobile phones facilitates the development of mobile health (mHealth)^26^. According to a recent survey, 83% of physicians in the U.S. use mobile health technology or mHealth to provide patient care^27^.

Different from a search engine, social media (e.g., Weibo and Twitter) can also provide a platform allowing people to share their personal experience and understanding of events, offering a new opportunity for public health practitioners to understand social and behavioural barriers to preventing infection. WPI’s performance may give earlier warning of H7N9 outbreaks than BSI, perhaps because Weibo is a platform of news reports and hot topics, while part of people’ search behaviour on Baidu following an event may generally lag behind the news and topics. With these real-time data, there are at least two potential directions for future research^28^. First, we can track the information on a given disease as it spreads across the social network as represented by Weibo. One study has found that public concern and engagement in protective behaviours increased when the threat of the H1N1 outbreak increased and decreasing when the perceived risk declined^29^. Second, content analysis of social networks posts will enable researchers to analyse human attitudes or reactions towards specific health hazards^30^. The data from social media may help identify individuals with anxiety or fears about infectious diseases who are not identified by the traditional clinical or survey approaches.

Recently, a prediction study for seasonal influenza integrated and analysed the human case data with Internet search surveillance data, meteorological data^31^, and human population data to improve the accuracy of the prediction model. Furthermore, Guo and his colleagues performed and assessed several forecasting models to track dengue fever disease dynamics, which achieved near real-time estimations of dengue incidence^32^. Tracking spatial and temporal trends on social media data, such as Facebook, Twitter and Weibo, can be applied to detect disease patterns, but estimating the potential time course and the geographic areas influenced by the disease remains challenging^33^.

There were some limitations of this study. First, the latitude and longitude of the province were replaced by the latitude and longitude of the provincial capital rather than geographic centre of the province in the linear regression model for BSI spatial analysis, because the capital city usually has the highest population density making it more representative. Second, the national geographical distribution of the WPI is not yet available, and it has not been included in this study. This study only describes the temporal and spatial consistency between H7N9 cases and the Internet search query results and explores their predictability. In the future, more use of visualization techniques to present the predictive model by using a variety of big data with verification set, such as an HTML document or Weibo thread or WeChat application, is warranted. This will largely increase the visibility of the study results and benefit a larger population of readers and researchers. With the development of new media and the shift of concerns, many other social media platforms such as WeChat may become a focus of future research, but the current limitation is the accessibility of the user data.

## Method

### H7N9 Cases, Internet search query data and social media data collection

In this study, we collected weekly laboratory-confirmed H7N9 cases reported in China during 1^st^ January 2013–31^st^ December 2017 from the Chinese National Influenza Centre (http://www.chinaivdc.cn/cnic/zyzx/lgzb/), the Department of Health of Hong Kong (http://www.dh.gov.hk) and the World Health Organization (WHO) (http://www.who.int/influenza/). We extracted data on each case’s basic information (including sex, age, location and date of disease confirmation) and clinical outcome. The first epidemic wave was from Jan 1^st^ to Sept 30^th^, 2013, and the subsequent three waves were from Oct 1^st^, 2013 to Sept 30^th^ of 2014, Oct 1^st^, 2014 to Sept 30^th^ of 2015, and Oct 1^st^, 2015 to Sept 30^th^ of 2016. The last wave was from Oct 1^st^, 2016 to Dec 31^st^, 2017^2^.

Baidu is the most popular internet search tool in China^14^. The Baidu Search Index (BSI) (http://index.baidu.com) makes available search volumes for different search terms using the Baidu search engine beginning in June 2006. The BSI is available at various spatial and temporal levels, including municipal, provincial or national spatial scale and daily, weekly, monthly or yearly time scale. Sina Weibo (hereafter ‘Weibo’) is the most widely used Microblog in China^28^, where users can share information and communicate with each other instantly. The Weibo Post Index (WPI) (http://data.weibo.com/index) is a composite index which incorporates the posting volume, reading volume and searching volume of a popular term.

In this study, “H7N9”, “Avian influenza” (禽流感 in Chinese) and “Live poultry” (活禽 in Chinese) were used search terms. “Symptom of H7N9” (H7N9 症状 in Chinses) and “Latest news on avian influenza for H7N9” (H7N9 禽流感最新消息 in Chinese) were also highly relevant to H7N9, but search results using the search term“H7N9” include the search results using “Symptom of H7N9” and “Latest news on avian influenza for H7N9”. Therefore, “H7N9”, “Avian influenza” and “Live poultry” were selected as the keywords to gather the BSI and WPI data. In this study, weekly data on BSI and WPI from personal computers (PC) and mobile phones between January 2013 and December 2017 were collected. Data on BSI index were available at the provincial level, but the WPI data were not, so only national data were used for WPI.

### Spatial and temporal description analysis

We mapped the distributions of BSI for “H7N9” at the provincial level. Seasonal decomposition analysis, a method to describe the systematic seasonal trends of a time series, was used to explore the seasonal variations of the H7N9 case number, BSI and WPI. The Poisson linear regression model was performed to assess the spatial dispersion between cases number and BSI with coordinates (longitude and latitude).

Comparisons of annual outbreak duration and peak time in five epidemic waves were conducted respectively using H7N9 case number, and BSI and WPI for H7N9-related terms. The start time of outbreak in each wave is defined as the number of weekly cases exceeding the median of all cases in each wave, and the end time of outbreak in each wave is when the number of weekly cases is below (and exceeds one week) the median value in each wave. These definitions were also used for BSI and WPI.

### Predictive model analysis

The cross-correlation analysis was used to measure the correlation between case number and BSI or WPI as a function of the displacement of one variable relative to the other, and to determine the time lag between two variables. After calculating the cross-correlation between the two variables, the lag corresponding to the maximum cross coefficient indicates the greatest correlation lag used in further analysis.

Seasonal autoregressive integrated moving average (SARIMA) model was developed to predict H7N9 cases using BSI and WPI data. We used the H7N9 case number as the dependent variable, and BSI and WPI with the maximum cross-correlation coefficient as the independent variables. An autoregressive notation (p), a differencing notation (d) and a moving average notation (q) formed the multiplicative process of ARIMA as (p, d, q), and a seasonal autoregressive notation (p), a seasonal differencing notation (d) and a seasonal moving average notation (q) formed the multiplicative process of SARIMA as (p, d, q)^34^. A SARIMA model can be considered a good model if it has a large stationary R square (R^2^) value and a small Bayesian Information Criteria (BIC).

Classification and regression trees (CARTs) are non-parametric statistical methods. The independent variable can be a categorical (classification tree) or a continuous variable (regression tree)^35^. In this study, regression tree analyses were performed to determine the threshold effects of the hierarchical relationship of the weekly H7N9 case number with the weekly BSI and WPI. BSI and WPI with the maximal cross-correlation coefficient were segmented into subsets that were most likely to be associated with weekly H7N9 cases number^31,36^.

All data analyses and graphical maps were conducted by Origin Pro 8.0 and ArcMap 10.6 (version 10.6, ESRI Inc.); Cross-correlation and linear regression model were performed by SPSS version 25.0. (SPSS Inc.: Chicago, IL, USA). Regression tree and SARIMA model were conducted by R software version 3.4.3. Statistical significance was set at *P* < 0.05 (two-tailed test).

## Supplementary information

supplementary materials
