# Supplementary material for: Avian Influenza A (H7N9) and related Internet search query data in China
Source: Sci Rep. 2019 Jul 18;9:10434. doi: 10.1038/s41598-019-46898-y (PMC6639335; doi:10.1038/s41598-019-46898-y)
Supplement: Supplementary file 1 — supplementary materials [file 41598_2019_46898_MOESM1_ESM.pdf]

Supplementary materials

Avian Influenza A (H7N9) and related Internet search query data in China

Ying Chen[1, 2], Yuzhou Zhang[2], Zhiwei Xu[2], Xuanzhuo Wang[1], Jiahai Lu[1]*, Wenbiao Hu[2]*

Correspondence: Jiahai Lu (E-mail: lujiahai@mail.sysu.edu.cn) or Wenbiao Hu (E-mail: w2.hu@qut.edu.au).

[1] School of Public Health, Sun Yat-sen University, Guangzhou, China.

[2] School of Public Health and Social Work, Institute of Health and Biomedical Innovation, Queensland University of Technology, Brisbane, Australia.

Table S1. Poisson regression model of longitude and latitude with H7N9 cases and BSI from wave 1 to wave 5.

	Longitude				Latitude			
	Coefficients	Standard error	Wald Chi-square	P value	Coefficients	Standard error	Wald Chi-square	P value
Cases_W1	0.372	0.0545	46.591	<0.001	0.076	0.0275	7.686	0.006
Cases_W2	0.102	0.0188	29.238	<0.001	-0.023	0.0118	3.763	0.052
Cases_W3	0.023	0.0122	3.599	0.058	-0.036	0.0132	7.484	0.006
Cases_W4	0.102	0.0394	6.745	0.009	-0.046	0.0251	3.282	0.07
Cases_W5	0.065	0.0079	66.823	<0.001	-0.032	0.0072	19.82	<0.001
BSI_W1	0.051	0.0017	896.453	<0.001	-0.009	0.0018	24.038	<0.001
BSI_W2	0.044	0.0018	584.187	<0.001	-0.025	0.002	163.515	<0.001
BSI_W3	0.037	0.0023	258.582	<0.001	-0.018	0.0026	46.191	<0.001
BSI_W4	0.033	0.0021	247.083	<0.001	0.002	0.0025	0.746	0.388
BSI_W5	0.011	0.0014	69.394	<0.001	-0.005	0.0018	8.602	0.003

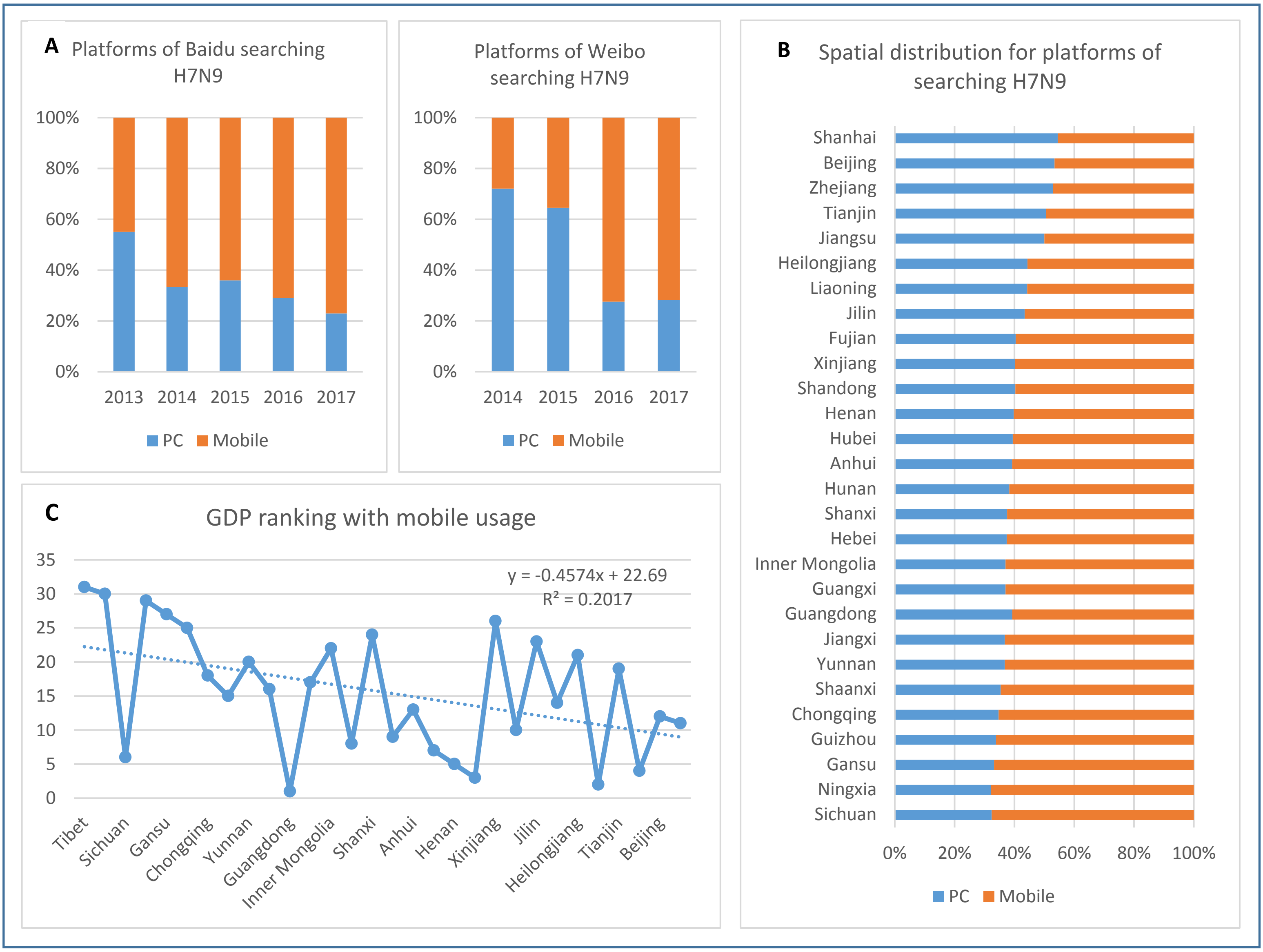

Figure S1. Using two platform of personal computer (PC) and mobile for H7N9 searching and posting based on Baidu Searching Index (BSI) from 2013-2017.

A: Time trend of using mobile phones or PC on Weibo and Baidu searching platforms. B: Spatial distribution in whole country for H7N9 searching using PC and mobile. C: The y-axis is the ranking of GDP, and the X-axis is the provinces sorted according to the proportion of mobile phone usage from high to low.

Table S2. Duration of H7N9 case outbreak start date and peak date with BSI and WPI for H7N9.

		W1	W2	W3	W4	W5
**H7N9 Cases**	Median	3	4	5.5	4.5	11
	Week count	11	33	24	28	46
	Outbreak start date	8-Apr	6-Jan	22-Dec	11-Jan	12-Dec
	Outbreak end date	6-May	21-Apr	9-Mar	28-Mar	15-May
	Duration of outbreak in weeks	5	16	12	14	23
	Peak date	22-Apr	3-Feb	2-Feb	8-Feb	30-Jan
	Peak value	39	50	27	10	63
		**W1**	**W2**	**W3**	**W4**	**W5**
**BSI**	Median	3862	1850	1235	978	937
	Week count	27	52	52	52	65
	Duration of outbreak in weeks	11	32	27	19	30
	Outbreak start date (D)	1-Apr (-1)	9-Dec (-4)	10-Nov (-6)	21-Dec (-3)	26-Dec (2)
	Outbreak end date (D)	10-Jun (5)	26-May (5)	11-May (9)	25-Apr (4)	17-Jul (9)
	Peak date (D)	15-Apr (-1)	27-Jan (-1)	19-Jan (-2)	11-Jan (-4)	27-Feb (4)
	Peak value	287846	27873	5810	14901	42163
		**W1**	**W2**	**W3**	**W4**	**W5**
**WPI**	Median	6634	1979	337	103	88
	Week count	26	52	52	52	65
	Outbreak start date (D)	8-Apr (0)	2-Dec (-5)	1-Dec (-3)	28-Dec (-2)	26-Dec (2)
	Outbreak end date (D)	10-Jun (5)	24-Mac (1)	4-May (8)	25-Apr (4)	3-Jul (7)
	Duration of outbreak in weeks	10	22	23	18	28
	Peak date (D)	8-Apr (-2)	17-Feb (2)	19-Jan (-2)	18-Apr (10)	20-Fec (3)
	Peak value	562733	91379	8688	2126	15428

Note: median indicates number of wave case or index.

D indicates the difference for date between H7N9 cases and BSI, WPI, respectively.

Table S3. Parameters estimates and their testing results of the SARIMA model (1,0,0)(1,0,0).

	Coefficients	Standard error	t	P value	R^2	BIC
**AR**	0.855	0.032	26.447	**<0.001**	0.736	3.585
**SAR**	0.125	0.077	1.632	0.104		
**BSI**	0.008	0.002	3.553	**<0.001**	0.748	3.550
**WPI**	0.002	0.001	2.103	**0.036**	0.740	3.581

A

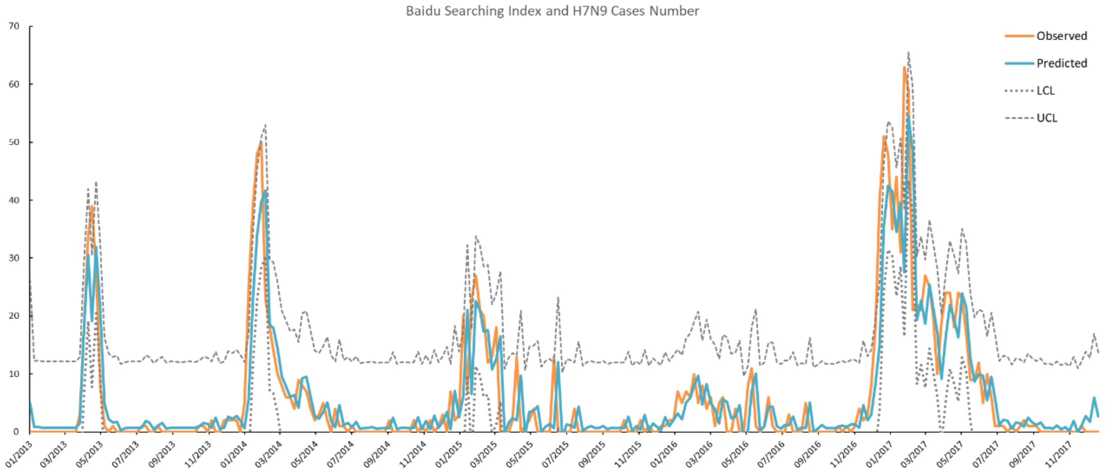

B

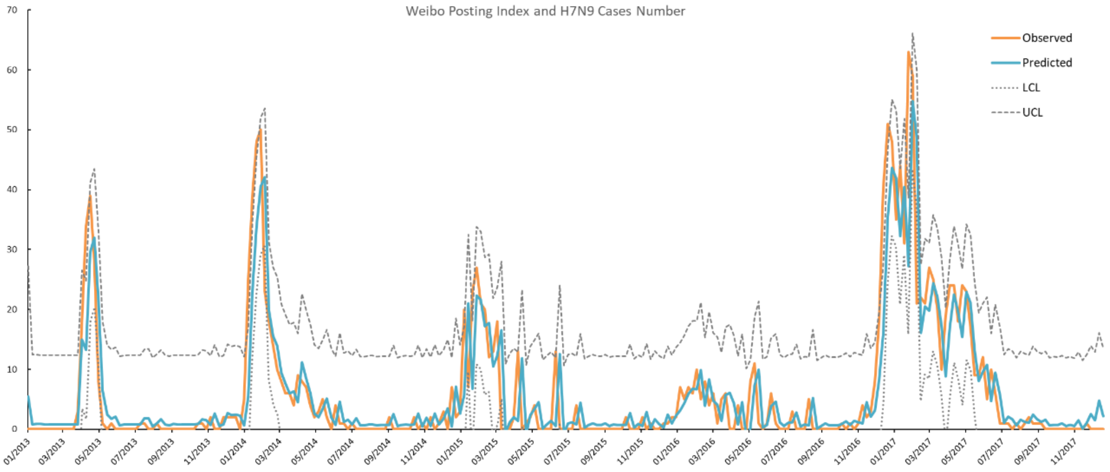

Figure S2: (A) Observed and fitted value of the ARIMA model (1, 0, 0) for H7N9 cases with Baidu Searching Index from 2013 to 2017. (B) Observed and fitted value of the ARIMA model (1, 0, 0) for H7N9 cases with Weibo Posting Index from 2013 to 2017. (Y axis: H7N9 cases number, X axis: date, UCL: upper control limit and LCL: lower control limit.)

a. The SARIMA model (1,0,0)(1,0,0) of BSI

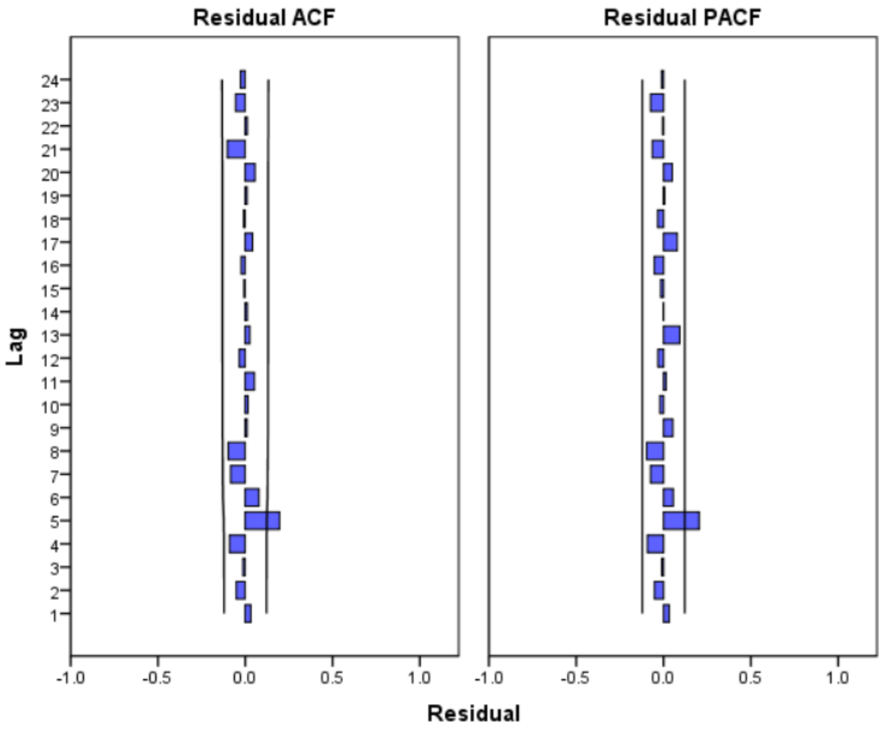

b. The SARIMA model (1,0,0)(1,0,0) of WPI

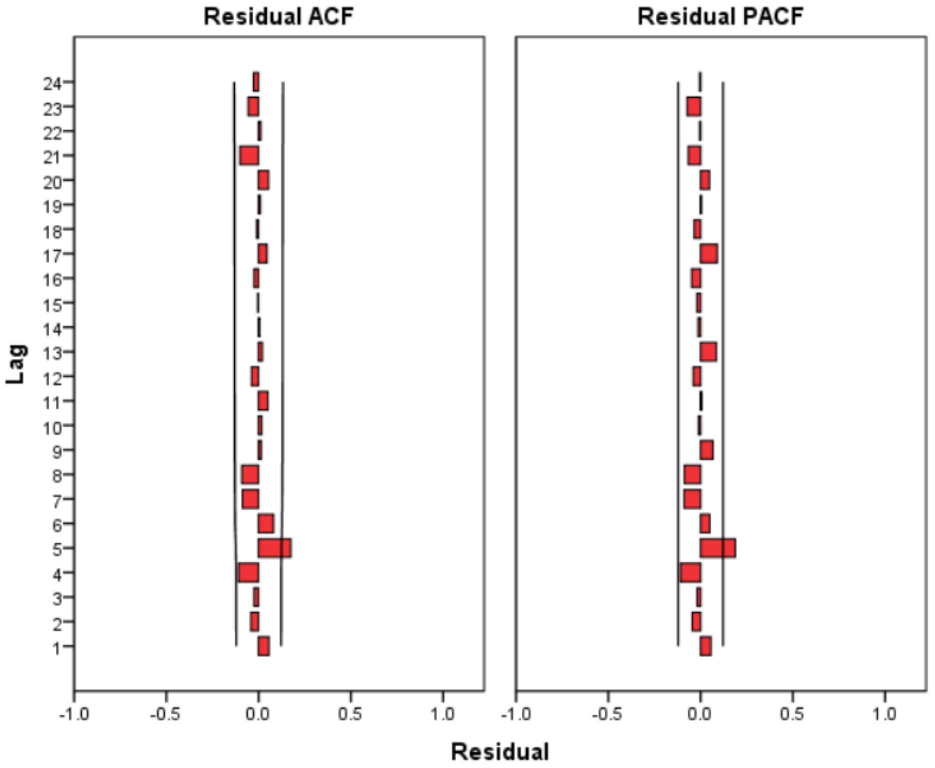

Figure S3. Autocorrelation function (ACF) and partial autocorrelation function (PACF) of the residual series of the SARIMA models
